# Occurrence of Entomopathogenic Fungi from Agricultural and Natural Ecosystems in Saltillo, México, and their Virulence Towards Thrips and Whiteflies

**DOI:** 10.1673/031.011.0101

**Published:** 2011-01-07

**Authors:** Sergio R. Sánchez-Peña, Jorge San-Juan Lara, Raúl F. Medina

**Affiliations:** ^1^ Departamento de Parasitología Agrícola, Universidad Autónoma Agraria Antonio Narro, Saltillo, Coahuila, México; ^2^ Department of Entomology, Texas A&M University, College Station, TX 77843-2475, USA

**Keywords:** habitat, germplasm, insect-pathogenic fungus, Hemiptera, Thysanoptera

## Abstract

Entomopathogenic fungi were collected from soil in four adjacent habitats (oak forest, agricultural soil, pine reforestation and chaparral habitat) in Saltillo, México using the insect bait method with *Tenebrio molitor* (L.) (Coleoptera: Tenebrionidae) larvae as bait. Overall, of the larvae exposed to soil, 171 (20%) hosted *Beauveria bassiana* (Balsamo) Vuillemin (Hypocreales: Cordycipitaceae), 25 (3%) hosted *Metarhizium anisopliae* (Metschnikoff) Sorokin (Hypocreales: Clavicipitaceae) and 1 (0.1%) hosted *lsaria* (=*Paecilomyces*) sp. (Hypocreales: Cordycipitaceae). *B*. *bassiana* was significantly more frequent on larvae exposed to oak forest soil. *M. anisopliae* was significantly more frequent on larvae exposed to agricultural soil. From the infected bait insects, 93 isolates of *B. bassiana* and 24 isolates of *M. anisopliae* were obtained. Strains were tested for their infectivity against Cuban laurel thrips, *Gynaikothrips uzeli* Zimmerman (Thysanoptera: Phlaeothripidae) and the greenhouse whitefly, *Trialeurodes vaporariorum* (Westwood) (Hemiptera: Aleyrodidae). *B. bassiana* isolates caused the highest mortality on thrips (some causing 88% mortality after 6 days); both fungal species caused similarly high mortality levels against whiteflies (75%) after 6 days. Large amounts of germplasm of entomopathogenic fungi, fundamentally *B. bassiana* and *M. anisopliae*, exist in the habitats sampled; pathogenicity varied among strains, and some strains possessed significant virulence. Soils in these habitats are reservoirs of diverse strains with potential for use in biocontrol.

## Introduction

Entomopathogenic fungi are distributed in a wide range of habitats including aquatic forest, agricultural, pasture, desert, and urban habitats (Sánchez-Pena 1990; Lacey et al. 1996; Chandler et al. 1997; Sánchez-Pena 2000). Their ability to regulate insect populations has been studied in tropical and temperate habitats (Evans 1982; Subinprasert 1987; Meyling and Eilenberg 2007). Soil is considered an excellent environmental shelter for entomopathogenic fungi since it is protected from UV radiation and other adverse abiotic and biotic influences (Keller and Zimmerman 1989). Fungal entomopathogens in the genera *Beauveria, Conidiobolus, Metarhizium* and *Isaria* (=*Paecilomyces*) are commonly found in soil (Domsch et al. 1980; Keller and Zimmermann 1989). The anamorphic entomopathogenic fungi *Beauveria bassiana* (Balsamo) Vuillemin and *Metarhizium anisopliae* (Metschnikoff) Sorokin (Hypocreales) are natural enemies of a wide range of insects and both fungi have a cosmopolitan distribution (Bidochka et al. 1998; Roberts and St. Leger 2004). These fungi can possibly also exist as saprophytes in soil.

The effects of factors such as geographical location, climatic conditions, habitat type, cropping system, and soil properties, as well as the effects of biotic factors on the occurrence and distribution of entomopathogenic fungi have been broadly studied (Chanler et al. 1997; Bidochka et al. 1998; Bruck 2004; Meyling and Eilenberg 2007; Quezada-Moraga et al. 2007). Consequently, to understand more about the insect-pathogen dynamics in the soil, studies on the natural occurrence, distribution and ecology of entomopathogenic fungi in different soil types and in different geographical regions are necessary (Bing and Xing 2008).

Much effort has been put into research on the development of *B. bassiana* and *M. anisopliae* as inundative biological control agents in agriculture and forestry in temperate regions. Recent advances in production, formulation, and application of fungal entomopathogens have resulted in rather well-known mycoinsecticide products based on *Lecanicillium lecanii* (Viégas), *Isaria fumosorosea* Wize (=*Paecilomyces fumosoroseus*) and *B. bassiana* (Hypocreales: Cordycipitaceae). These entomopathogenic fungi can suppress and provide good control of pests in greenhouse and field crops (Wraight et al. 2000) and several are currently being used or considered as commercial biocontrol agents. For instance, Ugine et al. (2005) showed that *B. bassiana* was virulent against western flower thrips, *Frankliniella occidentalis* exposed to Pergande (Thysanoptera: Thripidae) bean leaf foliage treated with conidia.

It is increasingly recognized that the biodiversity of agroecosystems delivers significant services, such as biological control of pests, to agricultural production (Meyling and Eilenberg 2007). The contribution of the entomopathogenic component of this biodiversity to the regulation of pest populations has often been ignored (Gurr et al. 2003) and when it has been acknowledged, it has usually been discussed if the introduction of exotic strains of fungi, or the augmentation of endemic strains, is an appropriate biocontrol strategy (Carruthers and Onsager 1993).

Projects in biological control of insects with fungi must consider the availability and acquisition cost of virulent strains from germplasm collections, and the regulation and legal restrictions to the transport and release of exotic entomopathogenic fungal strains outdoors. In this respect, the deployment of these fungi in agriculture is not straightforward, especially for developing countries. In order to circumvent these restrictions, it is important to explore local environments to detect promising, virulent fungal strains *in situ*.

The objectives of the present study were to explore diverse habitats as potential sources of local strains of entomopathogenic fungi virulent against important pests. Specifically, this study aims to explore the distribution of entomopathogenic fungi in soils from four adjacent habitats in the Chihuahuan desert of Saltillo, México, and to evaluate the virulence of some of these strains against selected insect hosts: whiteflies and thrips.

## Methodology

### Study site and collections of soil samples

The study was conducted at localities in the municipality of Saltillo, Coahuila, Mexico. This area is located in the southeastern state of Coahuila, centered at 25°31′ N, 101°37′ W, 1600 m above sea level (MASL). Soil classification follows that by the Government of Saltillo (2008). Soil samples were collected February, 2008 and March, 2008 in four ecosystems:

1. Forest of the endemic oaks, *Quercus taeda* Liebmann and *Quercus saltillensis* (Trelease) (Fagales: Fagaceae) at 25° 21′ 12.85″ N, 100° 59′ 39.11″ W, 1962 MASL; the soil type was regosol.

2. Agricultural soil (plots of pepper (*Capsicum anuum* L.), tomato (*Solanum lycopersicon* L.); also cabbage (*Brassica oleracea* var. *viridis* L.), prickly pear (*Opuntia ficus-indica* (L.) Mill.), wheat (*Triticum aestivum* L.) and corn (*Zea mays* L.)) located at 25° 21′ 24.61″ N, 101° 02′ 16.25″ W, 1749 MASL; the soil type was xerosol.

3. Chaparral of creosote bush (*Larrea tridentata* (Sessé & Mociño ex DC.) Coville) (Zygophyllales: Zygophyllaceae), lechuguilla (*Agave lechuguilla* Torr). (Asparagales: Agavaceae), and catclaw (*Acacia* spp.) (Fabales: Fabaceae) located at 25° 20′ 53.56″ N, 101° 01′ 11″ W, 1834 MASL; the soil type was litosol.

4. Exotic pine reforestation (*Pinus halepensis* Miller) (Pinales: Pinaceae) located at 25° 20′ 15.87″ N, 101° 01′ 32.90″ W, 1870 MASL; the soil type was litosol (2008).

At each of the four habitats, 14 locations were randomly chosen including a minimum distance of 10 m between them. Five samples were taken around each location producing 70 soil samples/habitat and a total of 280 soil samples. Using hand shovels, soil samples (about 200 g) were collected at a depth of at least 5 cm until filling >90% of 500 ml plastic cups. Shovels were cleaned with 70% ethanol between points. In the laboratory 30 ml of nonchlorinated, purified water was added to soil samples. The samples were left standing for 30 minutes to let the soil sample absorb the water. After 30 minutes the insect baits (three larvae) were placed in the cups. Last and penultimate larval instars of the mealworm, *Tenebrio molitor* L. (Coleoptera: Tenebrionidae) were used. Larvae were pooled from the laboratory colony and larvae purchased at pet stores. Extensive observations (previous to and concurrent with these tests) of these larvae incubated in moist chambers indicated that they were free of fungal entomopathogens.

### Isolation of entomopathogenic fungi

Isolation of the entomopathogenic fungi was achieved using the *Galleria* bait method (Zimmermann 1986) modified for *T. molitor*. Three *T. molitor* larvae were added to each sample and incubated at 25° C ± 1 for 15 days; samples were examined at 7 and 15 days. Dead larvae with or without incipient, visible external fungal growth were washed with tap water and placed individually in covered plastic cups (20 ml) with high moisture provided by a moist cotton ball and incubated for up to 15 days at room temperature.

### Isolate selection and culture

After incubation, fungal growth (spores) on bait insects was transferred with a sterile microbiology loop to agar media (potato dextrose agar plates plus 1% yeast extract) (PDAY, BD Bioxon, www.bd.com). Isolates growing on PDAY were identified microscopically (Humber 1997) from the insects they were taken, and from cultures. The name code for isolates (entomopathogenic fungi) was based according to habitat: Oak forest (A), Agricultural soil (B), Pine reforestation (C), Chaparral (D); sample number within habitat (1,2,3,4,5…70); repetition (A–E) and larvae number (1,2,3); thus samples were labeled B18C2, D5E2, etc.

### Source of insects for bioassay

Whiteflies, *Trialeurodes vaporariorum* (Westwood) (Hemiptera: Aleyrodidae) were collected from mallow, *Malva sylvestris* (L.) (Malvales: Malvaceae), false dandelion *Pyrrohopappus* sp. (Asterales: Asteraceae), and tomato at greenhouses in the Universidad Autónoma Agraria Antonio Narro (UAAAN). Tomato plants were grown for whitefly production in the greenhouse at 20° C ± 2° C. Cuban laurel thrips, *Gynaikothrips uzeli* Zimmerman (Thysanoptera: Phlaeothripidae), adults were collected from their host plant, *Ficus benjamina* (L.) (Urticales: Moraceae) in the cities of Matamoros, Tamaulipas, and Monterrey, Nuevo León.

### Laboratory Bioassays
Fungal Isolates

All the isolates were cultured on PDAY, with no more than two transfers from the insect host at 25° C ± 1 for 15 days and a 12 h photophase under diffuse fluorescent light. Isolates showing clearly abundant sporulation on agar plates were selected for bioassays. Cultures were less than 60 days old when used. The *B. bassiana* isolates were B18C2, C14D, D40E1, B8C1, C2B1, C32B1, D17B1, and D20E. The *M. anisopliae* isolates were B62B2, D5E1, and D5E2. Different fungi were used for the two insect species based solely on availability of cultures in the laboratory. Conidia obtained on solid medium were removed with a sterile spatula, and suspended in sterile purified drinking water with Tween 80 at 0.02%. The suspension was filtered through three layers of t-shirt cotton cloth and adjusted to a concentration of 1×10^7^ conidia/ml, using a Neubauer haemocytometer. Control insects were immersed in Tween 80 at 0.02% in water. For bioassays, insects were immersed in spore suspensions (see below).

It was not possible to perform (viability) germination test on the conidial suspensions used in these experiments. However, fungal suspensions prepared from cultures as described consistently have at least 50% germination and usually >90% (Sánchez-Peña and Vázquez-Jaime 1996; Sánchez-Peña unpublished observations)

### Whiteflies

Nymphs of *T. vaporariorum* were exposed to conidia from three *B. bassiana* isolates (B18C2, C14D, D40E1) and three *M. anisopliae* isolates (B62B2, D5E1, D5E2). Each isolate (treatment) consisted of four repetitions and each repetition included 65 second-instar nymphs. Tomato leaves infested with whitefly nymphs were immersed for 5 seconds in suspensions of fungal spores and excess liquid was drained off. Control insects were immersed in Tween 80 at 0.02% in water, without spores. Inoculated nymphs on excised leaves were placed in Petri dishes with a moist cotton ball. Mortality of insects was assessed at 3, 5 and 6 days after inoculation, under the dissecting microscope.

Nymphs infected with *B. bassiana* can show different responses. They were identified by: a dehydrated, shriveled aspect with sparse mycelium colonization; abundant mycelium colonization; and/or red pigments produced by the fungal mycelium (Eyal et al. 1993; Sanchez-Peña and Vazquez-Jaime 1996; Sánchez-Peña 1997).

### Thrips

Six *B. bassiana* isolates (B8C1, B18C2, C2B1, C32B1, D20E and D17B1) were used. Due to lack of insects *M. anisopliae* was not tested against thrips. Each treatment (isolate) was replicated four times; each replicate included 20 Cuban laurel thrips adults. Adult thrips were picked up with a soft hairbrush and placed in a fungal suspension for less than 5 seconds, then quickly transferred with the brush (blotting excess free water first from insects) to 50 ml cups with a moist cotton ball. Each cup held 20 thrips. Insect mortality was assessed 3, 5, and 6 days after inoculation, under the dissecting microscope.

### Data analysis

The number of infected insects was compared among habitats by the χ^2^ test, using the number of infected insects in the agricultural (horticultural) habitat as baseline for comparisons with the habitats sampled (Pezzullo 2010). To assess the differences in insect mortality among fungal isolates analysis of variance (ANOVA) and post-hoc Tukey's mean comparisons were used for bioassay data. In these, control mortality was never greater than 20%; all data were corrected for control mortality using Schneider-Orelli's formula (Bakr 2010). Percentage mortalities were logtransformed before analysis. Data were analyzed using the software Statistica version 7.0 (Statsoft, www.statsoft.com).

## Results

From the soil samples, 93 isolates of *B. bassiana* and 25 isolates of *M. anisopliae* were isolated on potato dextrose agar (PDA) (each isolate from one infected insect).

The occurrence of *B. bassiana* and *M. anisopliae* in Saltillo, Coahuila, Mexico, confirms their reported widespread distribution in soils worldwide (Vänninen 1996; Bidochka et al. 1998; Roberts and St. Leger 2004; Rehner 2005).

The entomopathogenic fungi *B. bassiana* and *M. anisopliae* were common in the sampled areas, but showed marked differences in abundance across habitats. These fungi were retrieved from 198 (23.57%) of the 840 incubated larvae; *B. bassiana* was isolated from 171 (20.4%) of larvae, and *M. anisopliae* from 25 (3%) (Table 1).

There were significant differences in frequency of fungi (number of infected insects) among habitats (χ^2^ = 63.27, p< 0.001). Most of the infected insects were obtained from oak forest soil (51.4%), followed by chaparral (18.0%), agricultural soil (16.2%) and pine reforestation (7.1%) (Table 1). Individually, *B. bassiana* and *M. anisopliae* were significantly more abundant in oak forest soil and in agricultural soil respectively (p<0.001). *B. bassiana* was significantly more abundant than *M. anisopliae* in all habitats (p<0.001) with the exception of agricultural plots, where there were not significant differences in number among both fungi (Table 1). Across habitats, *M. anisopliae* was significantly most abundant in agricultural soil only (p<0.001).

**Table 1.**
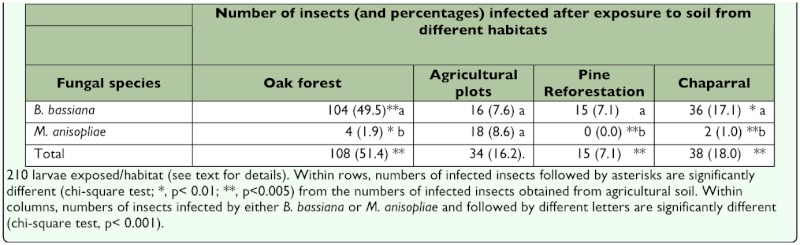
Distribution of entomopathogenic fungi as indicated by infected larvae exposed to soils from different habitats at Saltillo, Coahuila, Mexico, 2008.

In the bioassays against whitefly nymphs, there were essentially no significant differences among the fungi across isolates and species (Table 2). Out of the 3 *B. bassiana* isolates, isolate B18C2 caused 50% mortality (p<0.01) and 76% mortality after 5 and 6 days respectively (p<0.0001) (Table 1). *M. anisopliae* also caused significant mortality after 5 days. After 6 days mortality caused by all six isolates was again similar and highly significant (p<0.0001). Quesada et al. (2006) used 25 native *B. bassiana* isolates and a commercially available mycoinsecticide (based on *B. bassiana*); they found that all their isolates were pathogenic for two whitefly species (*Bemisia tabaci* and *T. vaporariorum*). In their work, mortality rates (8 days after inoculation) reached up to 85%.

**Table 2.**
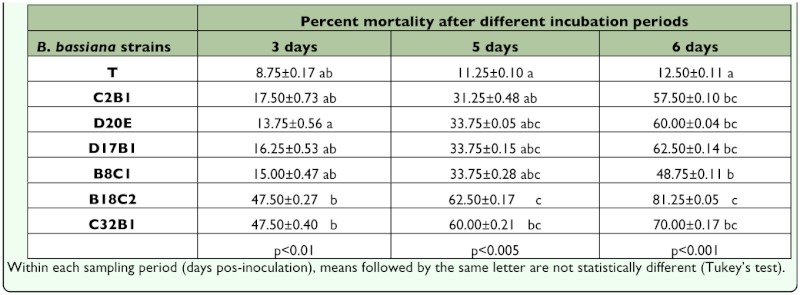
Percentage mortality ± SE at 3, 5 and 6 days in *Gynaikothrips ficorum* adults inoculated with Beauveria bassiana isolates at 1×107 conidia/ml.

On the other hand, there were rather large, significant differences in the mortality of thrips exposed to different strains of *B. bassiana* (Table 3). Out of the 6 *B. bassiana* isolates tested against *G. uzeli*, isolates B18C2 and C32B1 killed thrips fastest, causing the highest mortality after 3 days (47.5 %) (p<0.01). After 5 days, isolate B18C2 (the most active) caused 62.5% mortality (p<0.005); after 6 days, it caused 81.3% mortality (p<0.001); both values were significantly different from those of most isolates (Table 2). The second most active isolate against *G. uzeli* was C32B1 with 47.5 % mortality by 3 days (p<0.01), 60% mortality by 5 days (p<0.01) and 70% mortality by 6 days (p<0.001).

Some *B. bassiana* and *M. anisopliae* isolates did not cause significant mortality, at least initially; the relative activity of the isolates and the ranking of the mortality caused by them changed over time after inoculation in bioassays.

**Table 3.**
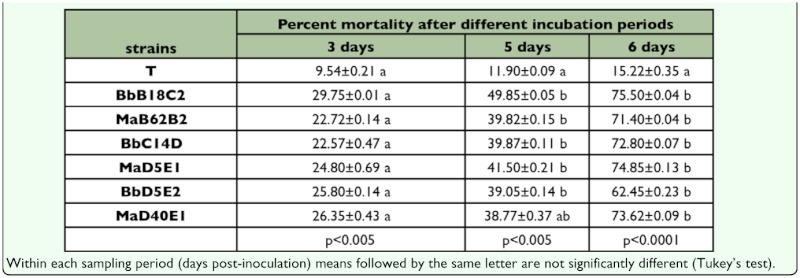
Percentage mortality (± SE) at 3, 5 and 6 days in 2nd-instar Trialeurodes vaporariorum nymphs inoculated with Beauveria bassiana (Bb) or Metharizium anisopliae (Ma) isolates at 1×107 conidia/ml

In general, there was overall variability in the pathogenic activity of the different isolates of *B. bassiana* and *M. anisopliae* obtained from the sampled habitats (Tables 2 and 3), particularly *B. bassiana* against thrips. *M. anisopliae* strains were rather homogeneous in their killing effect and there were not great differences on virulence against whitefly (*T. vaporariorum*) among isolates of *B. bassiana* and *M. anisopliae* (Table 3).

Not shown in the tables, but also recovered from larvae exposed in the oak forest were one isolate of the entomopathogenic fungus, *Isaria* sp. (=*Paecilomyces* sp.), and two species of hyperparasitic fungi including 3 specimens of *Syspastospora* sp. and 1 specimen of *Calcarisporium* sp., both parasitizing *B. bassiana*. Two larvae infected with the entomopathogenic nematode *Steinernema* sp. were also found.

## Discussion

Bing and Xing (2008) reported that *B. bassiana* and *M. anisopliae* often occurred in the soil of natural habitats and their presence was not significantly different between regions. However, the fact that *B. bassiana* is more frequent in natural habitats and *M. anisopliae* is more common in soils from cultivated habitats has been observed in several previous studies (Vänninen 1996; Bruck 2004; Quesada-Moraga et al. 2007). These authors reported no significant effect of habitat on the occurrence of *B. bassiana;* however, they found a strong association between *M. anisopliae* and the soils from cultivated habitats, particularly field crops. Unlike Quesada-Moraga et al. (2007), both fungi were never observed in the same sample in the present work. This difference can be due to the fact that Quesada-Moraga et al. (2007) exposed more larvae (ten vs. three); these authors also pooled five soil samples from each site before exposing insects to soil subsamples. In the present work, samples were not pooled, but used directly instead. This could have prevented mixing fungal propagules of different species in the soil sample that insects were exposed to. Quesada-Moraga et al. (2007) reported that 104 (42.6 %) and 18 (7.3 %) of soil samples harbored B. bassiana and M. anisopliae respectively, and 53 samples (21.7 %) harbored both species; in the present work, these two fungi were isolated from 171 (20.4%) larvae (B. bassiana) and from 24 (3%) (M. anisopliae) out of 840 larvae. Soil samples in Quesada-Moraga et al. (2007) and in this work were collected only once at each site; however, the occurrence of entomopathogenic fungi in the soil is likely to vary seasonally. Follow-up studies should determine if there is a seasonal effect on the occurrence of entomopathogens at this location, and compare those results with similar studies at other places.

Several studies suggest that *M. anisopliae* is more common in cultivated habitats, as was the case in the present work perhaps because *M. anisopliae* is relatively tolerant to pesticides (Quesada-Moraga et al. 2007; Brock 2009). The agricultural soils in the present work are routinely exposed to agricultural fungicides every growing season and year, and have been for decades. Vänninen et al. (2000) also found that *M. anisopliae* persisted much more than *B. bassiana* in different Finnish agricultural soils. Vänninen (1996) suggested that *B. bassiana* requires frequent serial passage through insects to survive, and that the relative scarcity of hosts in heavily cultivated (and sprayed) areas puts *B. bassiana* at a disadvantage in those soils. In contrast, *M. anisopliae* conidia are capable of longer-term persistence in the absence of arthropod hosts and have a higher survival in the soil than *B. bassiana* (Latch and Fallon 1976; Vanninen et al. 2000).

This study points out the importance of natural habitats as reservoirs of natural enemies for biological control purposes. Despite its apparent stability, the exotic pine reforestation was the poorest in terms of soil entomopathogens recovered. This may be an indication of the microbial community richness within reforested sites. Interestingly, the natural desert habitat had relatively high numbers of isolates (almost half as high as the oak forest habitat).

The presence of diverse fungal entomopathogens has been documented in tropical and temperate forests of Mexico and worldwide (Evans 1982; Sanchez-Peña 1990; Wongsa et al. 2005); these fungi are also producers of interesting metabolites (Wongsa et al. 2005).

These results show that considerable quantities of germplasm (strains) of entomopathogenic fungi exist in the Mexican habitats sampled, especially natural ones. This germplasm (fungal strains) is accessible for use in biological control of insect pests, locally or regionally. The fungi collected in this work include isolates with significant pathogenicity against the important pests tested (Hemiptera and Thysanoptera), and possibly against other insect pests as well, since there is no *a priori* reason to expect these isolates to show specific activity towards the tested insects. The differences in virulence observed among strains make it essential to evaluate fungal isolates before their use in biological control projects (Wraight et al. 2000).

In summary, the biodiversity, biocontrol, and natural products aspects make persuasive arguments supporting conservation of natural ecosystems and of biological resources like these fungi.

## References

[bibr01] Bakr E. M. (2010). LdP Line Software.. URL: http://www.ehabsoft.com/ldpline/..

[bibr02] Bidochka MJ, Kasperski JE, Wild GAM (1998). Occurrence of the entomopathogenic fungi *Metarhizium anisopliae* and *Beauveria bassiana* in soils from temperate and nearnorthern habitats.. *Canadian Journal of Botany-Revue Canadienne De Botanique*.

[bibr03] Bing DS, Xing ZL (2008). Occurrence and diversity of insect-associated fungi in natural soils in China.. *Applied Soil Ecology*.

[bibr04] Bruck DJ (2004). Natural occurrence of entomopathogens in Pacific Northwest nursery soils and their virulence to the black vine weevil, *Otiorhynchus sulcatus* (F.) (Coleoptera: Curculionidae).. *Journal of Environmental Entomology*.

[bibr05] Chandler D, Hay D, Reid AP (1997). Sampling and occurrence of entomopathogenic fungi and nematodes in UK soils.. *Applied Soil Ecology*.

[bibr06] Carruthers RI, Onsager JA (1993). Perspective on the use of exotic natural enemies for biological control of pest grasshoppers (Orthoptera: Acrididae).. *Environmental Entomology*.

[bibr07] Domsch KH, Gams W, Anderson TH (1980). Compendium of Soil Fungi..

[bibr08] Evans CH (1982). Entomogenous fungi in tropical forest ecosystems: an appraisal.. *Ecological Entomology*.

[bibr09] Eyal J, Mabud MDA, Fischbein KL, Walter JF, Osborne LS, Landa Z (1994). Assessment of *Beauveria bassiana* Nov. EO-1 strain, which produces a red pigment for microbial control.. *Applied Biochemistry and Biotechnology*.

[bibr10] Government of Saltillo. (2008). Información general..

[bibr11] Gurr GM, Wratten SD, Luna JM (2003). Multifunction agricultural biodiversity: pest management and other benefits.. *Basic Applied Ecology*.

[bibr12] Humber RA, Lacey LA (1997). Fungi: Identification.. *Manual of Techniques in Insect Pathology*.

[bibr13] Keller S, Zimmermann G, Wilding N, Collins NM, Hammond PM, Webber JF (1989). Mycopathogens of soil insects.. *Insect-Fungus Interactions*.

[bibr14] Lacey LA, Fransen JJ, Carruthers R, Gerling D, Mayer R (1996). Global distribution of naturally occurring fungi of Bemisia, their biologies and use as biological control agents.. *Bemisia: 1995. Taxonomy, biology, damage, control and management*..

[bibr15] Latch GCM, Fallon RE (1976). Studies on the use of *Metarhizium anisopliae* to control *Oryctes rhinoceros*.. *Entomophaga*.

[bibr16] Meyling NV, Eilenberg J (2007). Ecology of the entomopathogenic fungi *Beauveria bassiana* and *Metarhizium anisopliae* in temperate agroecosystems: Potential for conservation biological control.. *Biological Control*.

[bibr17] Pezzullo J. C. (2010). Two-way contingency table analysis at Statpages.. http://statpages.org/ctab2x2.html.

[bibr18] Quesada-Moraga E, Maranhao EAA, Valverde-García P, Santiago-Álvarez C (2006). Selection of *Beauveria bassiana* isolates for control of the whiteflies *Bemisia tabaci* and *Trialeurodes vaporariorum* on the basis of their virulence, thermal requirements, and toxicogenic activity.. *Biological Control*.

[bibr19] Quesada-Moraga E, Navas-Cortes JA, Maranhao EAA, Ortiz-Urquiza A, Santiago-Alvarez C (2007). Factors affecting the occurrence and distribution of entomopathogenic fungi in natural and cultivated soils.. *Mycological Research*.

[bibr20] Roberts DW, Leger RJ (2004). *Metarhizium* spp., cosmopolitan insect-pathogenic fungi: Mycological aspects.. *Advances in Applied Microbiology*.

[bibr21] Sánchez-Peña SR (1990). Some insect- and spider-pathogenic fungi from Mexico with data on their host range.. *Florida Entomologist*.

[bibr22] Sánchez-Peña S. R., Vázquez- Jaime G. (1996). Mortalidad acumulada tras aplicaciones sucesivas de tres hongos entomopatógenos sobre ninfas de mosca blanca.. Memorias XIX Congreso, Sociedad Mexicana de Control Biológico, Culiacán.

[bibr23] Statsoft, Inc. (2004). STATISTICA (data analysis software system)..

[bibr24] Subinprasert S (1987). Natural enemies and their impact on overwintering codling moth populations (*Laspeyresia pomonella* L.) (Lep., Tortricidae) in South Sweden.. *Journal of Applied Entomology*.

[bibr25] Ugine TA, Wraight SP, Brownbridge M, Sanderson JP (2005). Development of a novel bioassay for estimation of median lethal concentrations (LC50) and doses (LD50) of the entomopathogenic fungus *Beauveria bassiana*, against western flower thrips, *Frankliniella occidentalis*.. *Journal of Invertebrate Pathology*.

[bibr26] Vänninen I (1996). Distribution and occurrence of four entomopathogenic fungi in Finland: Effect of geographical location, habitat type and soil type.. *Mycological Research*.

[bibr27] Vänninen I, Tyni-Juslin J, Hokkanen H (2000). Persistence of augmented Metarhizium anisopliae and Beauveria bassiana in Finnish agricultural soils.. *Biocontrol*.

[bibr28] Wongsa P, Tasanatai K, Watts P, Hywel-Jones N (2005). Isolation and in vitro cultivation of the insect pathogenic fungus *Cordyceps unilateralis*.. *Mycological Research*.

[bibr29] Wraight SP, Carruthers RI, Jaronski ST, Bradley CA, Garza CJ, Galaini-Wraight S (2000). Evaluation of the entomopathogenic fungi *Beauveria bassiana* and *Paecilomyces fumosoroseus* for microbial control of the silverleaf whitefly, *Bemisia argentifolii*.. *Biological Control*.

[bibr30] Zimmermann G (1986). The “*Galleria* bait method” for detection of entomopathogenic fungi in soil.. *Journal of Applied Entomology*.

